# A Spontaneous Deletion of the *US1.67/US2* Genomic Region on the Bovine Herpesvirus 1 Strain Cooper

**DOI:** 10.1128/genomeA.01665-15

**Published:** 2016-02-04

**Authors:** F. S. Campos, W. P. Paim, A. G. Silva, R. N. Santos, R. M. Firpo, C. M. Scheffer, F. Finoketti, A. C. Franco, P. M. Roehe

**Affiliations:** Laboratório de Virologia, Instituto de Ciências Básicas da Saúde, Universidade Federal do Rio Grande do Sul, Porto Alegre, Rio Grande do Sul, Brazil

## Abstract

Bovine herpesvirus 1 (BoHV-1) is an alphaherpesvirus with a genome of 135 kb. Some BoHV-1 genes are nonessential and may be deleted from the viral genome. Here, a spontaneous gene deletion was identified in the BoHV-1 strain Cooper. Genes of the *US1.67/US2* region were absent, as determined by next-generation sequencing.

## GENOME ANNOUNCEMENT

Viruses are continuously undergoing genetic adaptations, where mutations and recombination are major players (1). These can be exemplified by genome rearrangements detected in laboratory-adapted strains of human cytomegalovirus (HCMV) (2). This may be of particular relevance for vaccine strains, which are often subjected to undefined number of passages *in vitro*. In the worst scenario, genetic alterations may lead to changes in genomic composition that may alter the immunizing capacity of a particular vaccine virus.

Here, the detection of a spontaneous deletion of two genes of the genome of a “classical” bovine herpesvirus type 1 (BoHV-1) vaccine strain (NVSL BoHV-1.1 Cooper reference strain) (3) is reported. The virus had been submitted to 12 successive passages in cell culture in our laboratory before sequencing. Virus multiplication was carried out in Madin-Darby bovine kidney cells and concentrated by ultracentrifugation, and DNA was extracted by usual methods (4). Sequencing of the whole viral genome was performed by next-generation sequencing in a MiSeq (Illumina) platform. A total of 645,622 raw reads were generated, with an average length of 139 bp, i.e., a coverage of 674× the whole BoHV-1 genome. Reads were trimmed using the Geneious software. After mapping, the viral genome revealed 99.1% identity to the reference strain Cooper, except for the lack of an 892-bp segment corresponding to the *US1.67/US2* genes.

The virus is believed to have undergone spontaneous deletion during *in vitro* passages. This finding provides additional evidence that, eventually, nonessential BoHV-1 genes may be deleted from viral genomes (5). In this case, no apparent prejudice for viral multiplication was noticed. Because BoHV-1 Cooper is a vaccine strain, the need for constant monitoring of the genomes of vaccinal viruses must be stressed, since undesired deletions may alter the immunogenic properties of the end product. This highlights the importance of strict control, including full-genome sequencing, over *in vitro* multiplication of viruses destined to vaccine production.

### Nucleotide sequence accession number.

The GenBank accession number is KU198480.

## References

[1] Baron S 1996 Medical microbiology, 4th ed. University of Texas Medical Branch at Galveston, Galveston, TX. 21413252

[2] Sijmons S, Ranst MV, Maes P 2014 Genomic and functional characteristics of human cytomegalovirus revealed by next-generation sequencing. Viruses 6:1049–1072. doi:10.3390/v6031049. 24603756 PMC3970138

[3] Zucheck F, Chow TL 1961 Immunogenicity of 2 infectious bovine rhinotracheitis vaccines. J Am Vet Med Assoc 139:236–237. 13788986

[4] Sambrook J, Russell DW 2001 Molecular cloning: a laboratory manual, 3rd ed. Cold Spring Harbor Laboratory Press, Cold Spring Harbor, NY.

[5] Rijsewijk FAM, Verschuren SBE, Madic J, Ruuls RC, Renaud P, van Oirschot JT 1999 Spontaneous BHV1 recombinants in which the gI/gE/US9 region is replaced by a duplication/inversion of the US1.5/US2 region. Arch Virol 144:1527–1537. 10486108 10.1007/s007050050608

